# Could Fabry Disease Cause Giant Coronary Aneurysms in a 7‐Month‐Old Infant: A Case Report

**DOI:** 10.1002/ccr3.73054

**Published:** 2026-07-01

**Authors:** Reza Shabanian, Minoo Dadkhah, Rana Doroudian, Mehrnaz Adib, Sahar Naderi, Khashayar Ashkboos

**Affiliations:** ^1^ Department of Pediatric Cardiology, Hakim Children's Hospital Tehran University of Medical Sciences Tehran Iran; ^2^ Department of Pediatric Endocrinology, Hakim Children's Hospital Tehran University of Medical Sciences Tehran Iran; ^3^ Department of Pediatric Rheumatology, Hakim Children's Hospital Tehran University of Medical Sciences Tehran Iran; ^4^ Department of Medicine Tehran University of Medical Sciences Tehran Iran

**Keywords:** coronary artery aneurysms, Fabry disease, Kawasaki disease, prolonged fever

## Abstract

Fabry disease is a rare X‐linked lysosomal storage disorder that can affect multiple organs. Cardiac involvement, one of its significant manifestations, can begin in childhood and is more prevalent in males, with severity increasing with age and disease progression. The most common cardiac feature in childhood is left ventricular hypertrophy (LVH), a significant contributor to morbidity and early mortality in adulthood. Other cardiac complications in Fabry disease include atrial fibrillation, ventricular tachycardia, conduction abnormalities, valvular insufficiency, hypertension, and coronary artery disease (CAD). While life‐threatening cardiac symptoms are typically absent in children, accelerated atherosclerosis can lead to CAD in adults. No prior reports have documented coronary artery disease in pediatric Fabry disease patients. We present the case of a 7‐month‐old infant who was admitted with prolonged fever, elevated inflammatory markers, and giant coronary artery aneurysms. Initially diagnosed and treated as atypical Kawasaki disease, further investigations identified a GLA mutation, suggesting Fabry disease as a possible contributing factor.

## Key Clinical Message

Fabry disease can present in early infancy with giant coronary artery aneurysms, mimicking severe, atypical Kawasaki disease. Clinicians should consider inherited metabolic disorders in the differential diagnosis of infants with atypical or refractory Kawasaki‐like presentations, as early diagnosis is crucial for initiating therapy.

## 1 Introduction

Fabry disease (OMIM 301500) is a rare X‐linked multisystemic lysosomal storage disorder affecting glycosphingolipid metabolism. It results from a partial or complete deficiency of the lysosomal enzyme α‐galactosidase A (α‐Gal A) due to mutations in the GLA gene located on Xq22. This deficiency leads to the progressive accumulation of glycosphingolipids, specially globotriaosylceramide (GL3), within lysosomes of various cell types, resulting in diverse clinical manifestations. To date, over 1000 mutations in the GLA gene have been identified [1, 2, 3].

In Fabry disease, the accumulation of Gb‐3 stimulates immune mechanisms and leads to chronic low‐grade systemic inflammation. It seems that the buildup of Gb‐3 activates immune pathways, particularly Toll‐like receptors (TLRs) and CD1d, leading to inflammatory responses through immune cells. Gb‐3 accumulation can act as a damage‐associated molecular pattern (DAMP) or induce injured cells to produce DAMPs. Additionally, it can provoke the secretion of cellular adhesion molecules (CAMs) and cytokines, promoting a pro‐inflammatory effect on leukocytes and endothelial cells. As the inflammatory process becomes chronic, it contributes to the development of fibrosis in various tissues, primarily affecting the heart and kidneys [3].

Lysosomal involvement in Fabry disease likely begins as early as the fetal stage. However, the first symptoms typically emerge after 3 years of age, often sooner in males due to the X‐linked inheritance pattern. The presentation of the disease in heterozygous women can vary significantly, ranging from asymptomatic to severe symptom [3]. Although Fabry disease is a rare metabolic disorder, recent studies in newborns have revealed a higher incidence than previously reported. This suggests that the disease has likely been underdiagnosed [4, 5].

Males with Fabry disease typically experience symptoms earlier and more frequently than females. The median age of symptom onset is 6 years for males and 9 years for females. Given that FD involves multiple organs, it can lead to a variety of clinical symptoms. In childhood and teenage period, FD can present with one or more of these signs and symptoms: Acroparaesthesia, Neuropathic pain, Persistent “growing pains”, Recurrent unexplained headaches, Gastrointestinal symptoms, Hearing loss, Angiokeratoma, Dyshydrosis (hypo and hyperhidrosis), Heat intolerance, Unexplained microalbuminuria/proteinuria, Exercise intolerance and Fatigue, Recurrent unexplained fever, Depression, Ophthalmological involvement like Vortex keratopathy, Cataract and Conjunctival vessel tortuosity. Also, one of the most important manifestations of FD is cardiac involvement [1, 6].

FD may present only with manifestations limited to the heart that called cardiac type FD. Fabry disease phenotypes divided into two groups: classic and non‐classic. The classic phenotype presents with multi‐organ involvement during childhood or adolescence, with males affected earlier than females. On the other hand, the non‐classic phenotype tends to show clinical signs later in life, typically between the fourth and sixth decades, although symptoms can manifest in childhood with very different symptoms from those of the classic form. Adult‐onset and renal variants are more prevalent [7].

Cardiac involvement in FD accounts for 40%–60% of patients and can begin in childhood with slow progression. Cardiac symptoms have higher prevalence in males than in females and increase with age and disease progression. Due to deposition of Gb3 in myocytes, vascular endothelial cells, and valvular fibroblasts, the myocardium, conduction system, and valves may all be impacted. The most frequent cardiac manifestation is left ventricular hypertrophy (LVH), which can occur in childhood and is a major cause of morbidity and early mortality in adults. However, cardiomyopathy is commonly asymptomatic in the early stage of FD; cardiac symptoms as index presentation are in almost 10% of patients. In untreated individuals, LVH is present in 53% of males and 33% of females, increasing with age. Additionally, cardiac complications include atrial fibrillation, non‐sustained ventricular tachycardia, QRS broadening, short PR interval, atrioventricular block, sinus node dysfunction, valvular insufficiency, hypertension, and coronary artery disease. Life‐threatening cardiac symptoms are not reported in the pediatrics before the teenage years. Accelerated atherosclerosis in FD can lead to coronary artery disease, potentially causing myocardial infarction and death. This lipid‐driven process is distinct from the transmural inflammatory destruction that causes coronary aneurysms, as seen in Kawasaki disease. FD patients also often have traditional CAD risk factors, increasing their risk further. There are case reports related to coronary artery disease in patients with FD, but so far, there have been no reports of it in the pediatric population [1, 2, 6, 8, 9, 10, 11].

Herein we present a 7‐month‐old infant who admitted with prolonged fever and giant aneurysms of the coronary arteries. After being treated as Atypical Kawasaki disease, in further investigations confirmed a diagnosis of Fabry disease.

## 2 Case History/Examination

A 7‐month‐old male infant with an unremarkable previous medical history presented to our hospital after 21 days of fever. There was no history of conjunctivitis, rash, changes in the extremities, cervical lymphadenopathy, or mucosal membrane changes. The parents did not report any respiratory or gastrointestinal symptoms. Over the course of three weeks of fever, he was treated as an outpatient with oral antibiotics and antipyretic medications, but the fever remained uncontrolled. Laboratory tests were performed, which showed elevated inflammatory markers. Upon admission to our hospital, he had a fever and tachycardia. Blood pressure and respiratory rate were normal. On examination, aside from erythema of the pharynx, there were no other positive findings.

## 3 Differential Diagnosis, Investigations and Treatment

Initial laboratory tests showed anemia, thrombocytosis, elevated C‐reactive protein, and high erythrocyte sedimentation rate. Liver function tests, urinalysis, and blood and urine cultures were reported as normal. The main findings on his laboratory tests are shown in Table 1.

**TABLE 1:** The patient's laboratory test results.

Test	Result	Reference range
White blood cells	11.7	4–10 × 10^3^/uL
Neutrophil%	54	
Lymph%	39	
Mon%	5	
Eos%	2	
Hemoglobin	8	11–16 g/dL
Platelets	1018	150–450 × 10^3^/uL
ESR	130	0–10 mm/h
CRP	52	Negative: < 10
AST	35	< 40 U/L
ALT	26	< 40 U/L
Albumin	4.1	3.5–5.2 g/dL
Ferritin	215	7–140 ng/mL
c‐ANCA	6.6	< 10 U/ML
p‐ANCA	< 1	< 15 U/ML

Prior to admission, the infant had been evaluated at multiple outpatient clinics and a children's hospital. Repeated blood cultures, chest radiography, and urinalysis were negative. Despite empiric broad‐spectrum antibiotics, the fever persisted for three weeks, and no localized source of infection was identified, making an infectious etiology unlikely. Additionally, serologic tests for Epstein–Barr virus, cytomegalovirus, adenovirus, and parvovirus B19 were unremarkable, and autoimmune markers (ANA, anti‐dsDNA, ANCA) were negative.

**FIGURE 1:**
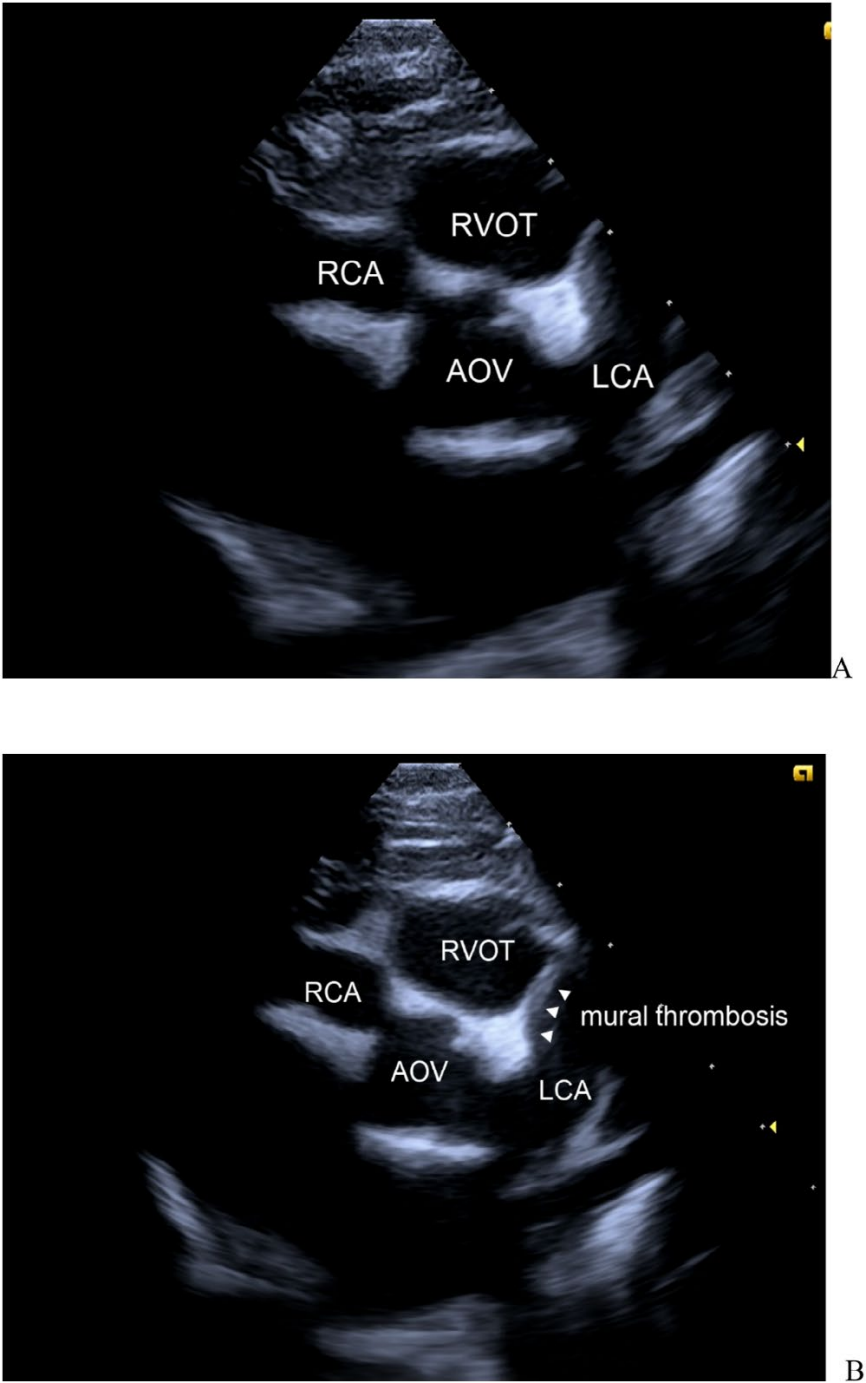
(A) Gigant aneurysm in both coronary arteries with mural thrombosis in left coronary arteries, (B) Mural thrombosis. The patient was treated as atypical Kawasaki disease with IVIG, methylprednisolone pulse, infliximab, aspirin, LMWH, and a beta‐blocker. AOV, aortic valve; LCA, left coronary artery; RCA, right coronary artery; RVOT, right ventricular outflow tract.

Echocardiography revealed fusiform and saccular aneurysmal formation in the RCA, LAD, and LCX, along with mural thrombosis (Figure 1). Chest x‐ray and abdominopelvic ultrasound were performed, both of which were normal.

## 4 Outcome and Follow‐Up

The patient's fever subsided 48 h later, another echocardiogram was performed, which showed that the thrombosis had resolved, but there was no change in the size of the aneurysms. Due to the unusual manifestation of the disease (3 weeks fever that did not match Kawasaki disease) and the consanguinity of the parents, we performed Whole Exome Sequencing (WES) to rule out genetic causes. WES revealed a mutation in the GLA(NM_000169.3) gene, variant c.427G > A (p.Ala143Thr) consistent with X‐linked Fabry disease. The level of alpha‐galactosidase enzyme was significantly low. The patient's mother also underwent genetic testing, which revealed that she is a carrier of the disease.

## 5 Discussion

Fabry disease (FD) represents a significant clinical challenge due to its complex pathophysiology and diverse clinical manifestations. Cardiac involvement in FD is well established; in 2016, Ellaway reported that the median age of symptom onset in males was 6 years. Our 7‐month‐old patient underscores the critical need for heightened awareness and early diagnosis of FD, even in atypical presentation [1].

The unresolved question in the present case is whether the observed giant coronary aneurysms represent a direct complication of Fabry disease, a coincidental Kawasaki spectrum, or an interaction between the two. For our patient, three possible scenarios deserve consideration, though none can be proven definitively. First, Fabry disease and atypical Kawasaki disease might have occurred independently; the infant, who had underlying Fabry disease, developed Kawasaki disease just like any other child. This scenario is somewhat unlikely, given the three‐week fever (longer than is typical for Kawasaki disease), the absence of other characteristic features, and the age of under 1 year. Second, the chronic low‐grade inflammation driven by Fabry disease could have predisposed the patient to a Kawasaki‐like systemic vasculitis, acting as a trigger. Third, the giant coronary aneurysms might represent a direct—though previously unreported—cardiac manifestation of Fabry disease in infancy. While these hypotheses are intriguing, the causal relationship between Fabry disease and the coronary phenotype remains speculative, and further evidence is required to clarify the underlying mechanism.

The type of our patient's mutation was c.427G > A (p.Ala143Thr). This mutation in the GLA gene is significantly associated with the cardiac variant of Fabry disease. Studies have shown that this mutation typically leads to primarily cardiac symptoms, such as left ventricular hypertrophy, with a later onset and milder overall disease severity compared to the classic form of Fabry disease. Patients with this mutation often exhibit reduced but residual alpha‐galactosidase A enzyme activity, contributing to a milder phenotype and predominantly cardiac manifestations. the genetic mutation present in our patient [12]. In a newborn screening study conducted by Lin et al. on the Taiwanese Chinese population, an increased incidence of the cardiac variant of Fabry's disease was observed. The study screened 16 males diagnosed with idiopathic hypertrophic cardiomyopathy using mutation analysis and α‐galactosidase activity. This combined approach revealed deficient enzyme levels and mutations in 4 cases (25%). The study identified the IVS4 + 919G → A mutation in both newborns and patients with idiopathic cardiomyopathy, which was different from the mutation reported in our patient [13].

Fabry disease exhibits a broad spectrum of symptoms due to its multisystemic nature. In males, symptoms often present earlier and with greater severity due to the X‐linked inheritance pattern. The cardiac symptoms in FD can be subtle in the early stages, often delaying diagnosis until more severe complications arise. However, it can occur in early ages as in 2019 Akidi reported a Rare Case of Cardiac Variant of Fabry's Disease in a 2‐Year‐Old Child [14]. Our patient, presenting with prolonged fever and giant coronary artery aneurysms, was initially suspected to have atypical Kawasaki disease. This case highlights the diagnostic challenge posed by FD, particularly in pediatric patients where symptoms may not align with more common pediatric conditions. The use of enzyme assay and Whole Exome Sequencing (WES) was pivotal in identifying the underlying genetic mutation in the GLA gene, confirming the genetic diagnosis of Fabry disease. Nevertheless, as Gragnaniello et al. discussed, there are several concerns, and newborn screening for Fabry disease has not yet been universally accepted [15].

Cardiac manifestations are a major cause of morbidity and mortality in FD. The deposition of GL3 in myocytes, endothelial cells, and fibroblasts leads to various cardiac complications, including LVH, arrhythmias, and valvular dysfunction. In childhood, cardiac issues associated with Fabry disease exhibit subtle changes that progress slowly but become detectable over time [16]. Left ventricular hypertrophy (LVH) is the predominant cardiac manifestation of Fabry disease making FD a significant consideration in the differential diagnosis of patients presenting with unexplained hypertrophic cardiomyopathy [17, 18, 19, 20]. In our case, the presence of giant coronary artery aneurysms with mural thrombosis is particularly noteworthy. While cardiac involvement typically progresses slowly and is often asymptomatic in early childhood, our patient's severe presentation underscores the variability and potential severity of FD cardiac pathology.

The case emphasizes the importance of considering Fabry disease in the differential diagnosis of pediatric patients with prolonged fever and atypical cardiovascular findings. Early and accurate diagnosis is crucial for initiating appropriate therapies, such as enzyme replacement therapy (ERT) or chaperone therapy, which can mitigate disease progression and improve quality of life. Genetic testing, including WES, plays a crucial role in identifying FD, especially in cases with atypical presentations and a family history suggestive of an inherited disorder.

## 6 Conclusion

The presentation of Fabry disease in this 7‐month‐old infant with prolonged fever and giant coronary artery aneurysms underscores the importance of early recognition and diagnosis of this rare metabolic disorder. While the co‐occurrence of Fabry disease and giant coronary aneurysms is striking, a causal relationship cannot be definitively established from a single case. Chronic inflammation, multi‐organ involvement, and significant cardiac manifestations necessitate a high index of suspicion and comprehensive diagnostic evaluation. This case highlights the need for increased awareness among clinicians to consider Fabry disease in similar presentations, ultimately leading to timely and effective management of this complex condition.

## Author Contributions

**Reza Shabanian:** conceptualization, investigation, methodology, validation, visualization, writing – original draft. **Minoo Dadkhah:** investigation, writing – review and editing. **Rana Doroudian:** investigation, writing – review and editing. **Mehrnaz Adib:** investigation, writing – review and editing. **Sahar Naderi:** conceptualization, methodology, project administration, supervision, writing – review and editing. **Khashayar Ashkboos:** investigation, writing – original draft.

## Funding

The authors have nothing to report.

## Consent

Written informed consent was obtained from the patient's legal guardians for the publication of this case report and any accompanying images.

## Conflicts of Interest

The authors declare no conflicts of interest.

## Data Availability

The data that support the findings of this study are available on request from the corresponding author. The data are not publicly available due to privacy or ethical restrictions.
